# Taking the STING Out of Ureteral Obstruction

**DOI:** 10.1089/cren.2016.0092

**Published:** 2016-10-01

**Authors:** Jack Crozier, Ivan Aw, Philip Huang Min Tan, David Clarke

**Affiliations:** ^1^ Department of Surgery, Western Health, Footscray, Australia.; ^2^ Department of Surgery, The University of Melbourne, Victoria, Australia.

**Keywords:** vesicoureteral reflux, STING, ureteral obstruction, Teflon, flank pain

## Abstract

Vesicoureteral reflux (VUR) is diagnosed in ∼1% of children. The main goal of treatment is preservation of renal function by preventing recurrent urinary tract infection (UTI) refractory to antibiotic therapy. Surgical treatment options include endoscopic injection or ureteral reimplantation. Subureteral Teflon (polytetrafluoroethylene) injection (STING) is an endoscopic treatment option no longer in common practice. Use of Teflon is no longer advised because of a number of documented complications secondary to local and distant migration of injected material. We present a case of delayed ureteral obstruction secondary to the STING procedure occurring 21 years after initial surgery and managed using a novel endoscopic method.

## Introduction and Background

Vesicoureteral reflux (VUR) is diagnosed in ∼1% of children.^1^ The main goal of treatment is preservation of renal function by preventing recurrent urinary tract infection (UTI) refractory to antibiotic therapy. Historically, most children with VUR receive prophylactic antibiotics regardless of severity of reflux, and surgical management is indicated in cases of breakthrough UTIs. Surgical treatment options include endoscopic injection or ureteral reimplantation. Subureteral Teflon (polytetrafluoroethylene) injection (STING) was first introduced by O'Donnell in 1984.^2^ O'Donnell's initial human trial included 14 participants and 18 ureters with varying degree of reflux.^2^ After the STING procedure, 17 ureters had complete absence of reflux. Endoscopic treatment not only approaches the success rates of open ureteral reimplantation but also offers significant advantages such as outpatient day procedure, lower morbidity, postoperative recovery time, and reduced cost. The procedure gained traction and became a first-line surgical treatment, but subsequent follow-up identified potentially serious adverse events arising from the use of Teflon. Local and distant Teflon migration and foreign body reaction (FBR) may result in ureteral obstruction. As a result, the technique was modified and use of Teflon is no longer advised.^3^ We present a case of delayed complication after the STING procedure using Teflon.

## Presentation of Case

In November 1992, a 17-year-old male underwent a bilateral STING procedure using Teflon for persistence of bilateral VUR. The VUR was effectively treated both clinically and radiologically and he remained asymptomatic until 13 years later, when he developed right-sided flank pain. Computer tomography with intravenous urogram (CT IVU) was performed, showing amorphic calcifications at the vesicoureteral junction (VUJ) bilaterally, and marked right hydronephroureter that extended down to the level of the VUJ.

**FIG. 1.**
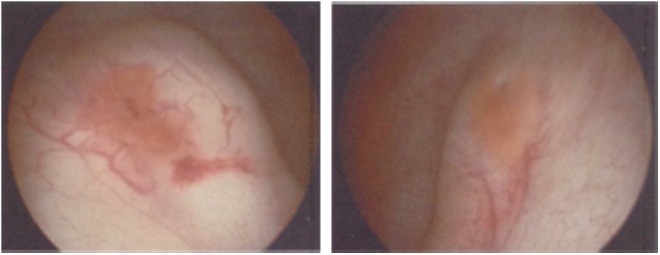
Cystoscopy findings: raised mucosa adjacent right and left ureteric orifice.

**FIG. 2.**
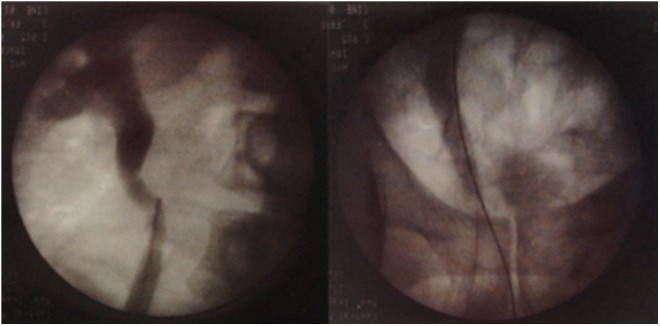
Right RPG showing hydroureteronephrosis. RPG, right retrograde pyelogram.

**FIG. 3.**
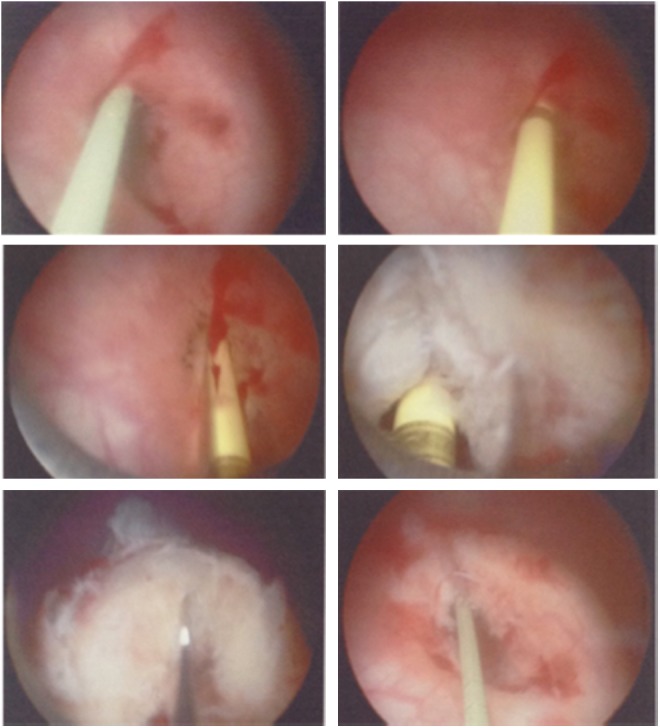
Sachse urethrotomy of right ureteral orifice.

During cystoscopy, raised erythematous mucosa adjacent to both the ureteral orifices was seen (Fig. 1) and right retrograde pyelogram (RPG) showed hydroureteronephrosis (Fig. 2). The right ureteral orifice was incised at the 12 o'clock position with a Sachse urethrotome (Fig. 3).

Repeat CT IVU at 3 months revealed no residual ureteral obstruction. The right kidney was shown to contribute 57% of renal function on dimercaptosuccinic acid scan. Decision was made to monitor the contralateral kidney as the patient was asymptomatic and had no radiographic evidence of obstruction.

**FIG. 4.**
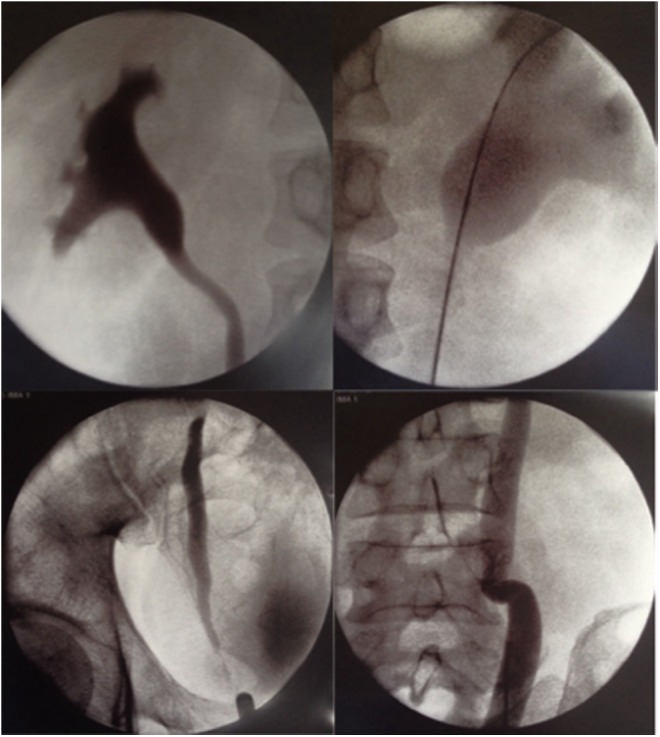
Comparison of right and left RPG shows obstruction limited to left side.

Seven years later (and 21 years after his initial surgery), the patient was readmitted to hospital with contralateral left flank pain. CT and ultrasonography confirmed left hydroureter and hydronephrosis down to the level of the VUJ. True to their original plan, his treating surgeon performed a left ureteral dilatation and endoscopic incision of the ureteral orifice with a Sachse urethrotome. Intraoperative bilateral RPG showed that hydroureteronephrosis was isolated to the left side, with a nondilated right collecting system (Fig. 4).

Mercaptoacetyltriglycine (MAG3) scan at 3 months showed no evidence of obstruction and near equal split renal function. More than 6 months after his surgery, the patient remains pain free.

## Discussion and Literature Review

To our knowledge, this is the first case of ureteral obstruction secondary to STING procedure definitively managed by endoscopic surgery. Prior studies have reported effective treatment with more invasive treatment using the Leadbetter-Politano ureteroneocystostomy technique.^4^ Our case also describes the longest delay to obstruction (21 years) after the STING procedure.^4^ Previous reports recorded a wide interval between STING and detection of ureteral obstruction from 1 to 168 months.^4^

Ureteral obstruction is a well-documented complication of the STING procedure. Although imaging may often show calcification around the VUJ mimicking stone, w**e** have not identified any cases of ureteral stone secondary to the STING procedure. More worrisome complications that can occur include possible migration of Teflon particles to distant organs including the brain and lungs, resulting in pulmonary embolism or cerebrovascular events.^3^

Surgical technique and location of Teflon injection were postulated as the main factor for FBR and migration. Although submucosal injection does not elicit marked reaction, injection into detrusor and perivesical fat may result in significant FBR and fibrosis. Although the incidence of acute obstruction is 0.7%–5.7%, and can be managed with a temporary stent or nephrostomy, delayed obstruction is rare and no risk factors have been identified to date.^5^

These adverse outcomes have led to a shift away from Teflon application in the pediatric population.^4^ New injectable agents such as dextranomer hyaluronic acid copolymer (Deflux) have been introduced in combination with the STING procedure and offer an 80% cure rate.^6^ Although Deflux appears to offer lower cure rates than Teflon, it offers a better safety profile as animal studies have failed to show distant migration using Deflux. The STING procedure has also been modified because of concern that bulking agents may migrate caudal to the ureteral orifice, thus rendering them ineffective and possibly resulting in long-term procedure failure.^7^ Initially, the procedure described by O'Donnell required injection of the bulking agent submucosally at the 6 o'clock position distal to the affected ureteral orifice.^7^ The most popular method described to increase procedure success is the hydrodistention implantation technique as defined by Kirsch et al.^8^ The Kirsch method involves a pressured stream of irrigation fluid directed into the refluxing ureter so that the ureter injection site could be defined. Then a needle is introduced ∼4 mm into the refluxing ureter at the 6 o'clock position.^7^

Factors associated with the STING procedure failure include younger age group (<54 months) and history of prior STING failure.^9^ The lower success rate in young children has been postulated to be caused by higher voiding pressures and unstable bladder dynamics in children still undergoing toilet training.^9^ It has been shown that variations in ureteral orifice configuration do not influence the short-term success rate of endoscopic surgery. Although endoscopic treatment of VUR may have lower treatment success rates than alternative open procedures, it remains the mainstay of first-line treatment as it is minimally invasive and has less complications.^10^

## Conclusion

We describe the first reported case of ureteral obstruction secondary to the STING procedure managed entirely endoscopically. Endoscopic treatment of VUR offers significant advantage of avoiding potentially complicated open surgery. Although our follow-up is short, it remains a reasonable treatment alternative as first-line treatment-reserving ureteral reimplantation for obstruction refractory to endoscopic treatment. Awareness of delayed ureteral obstruction should encourage longer follow-up for patients after the STING procedure.

## Abbreviations Used

CT: computed tomography
CT IVU: computer tomography with intravenous urogram
FBR: foreign body reaction
RPG: right retrograde pyelogram
STING: subureteral Teflon injection
UTI: urinary tract infection
VUJ: vesicoureteral junction
VUR: vesicoureteral reflux
